# Observer-Based Event-Triggered Predictive Control for Networked Control Systems under DoS Attacks

**DOI:** 10.3390/s20236866

**Published:** 2020-11-30

**Authors:** Weifan Lu, Xiuxia Yin, Yichuan Fu, Zhiwei Gao

**Affiliations:** 1 The Department of Mathematics, School of Science, Nanchang University, Nanchang 330031, China; luweifan1217@163.com; 2 The Faculty of Engineering and Environment, University of Northumbria at Newcastle, Newcastle upon Tyne NE1 8ST, UK; yichuan.fu@northumbria.ac.uk (Y.F.); zhiwei.gao@northumbria.ac.uk (Z.G.)

**Keywords:** event-triggered control, static observer, DoS attack, predictive control, compensation

## Abstract

This paper studies the problem of DoS attack defense based on static observer-based event-triggered predictive control in networked control systems (NCSs). First, under the conditions of limited network bandwidth resources and the incomplete observability of the state of the system, we introduce the event-triggered function to provide a discrete event-triggered transmission scheme for the observer. Then, we analyze denial-of-service (DoS) attacks that occur on the network transmission channel. Using the above-mentioned event-triggered scheme, a novel class of predictive control algorithms is designed on the control node to proactively save network bandwidth and compensate for DoS attacks, which ensures the stability of NCSs. Meanwhile, a closed-loop system with an observer-based event-triggered predictive control scheme for analysis is created. Through linear matrix inequality (LMI) and the Lyapunov function method, the design of the controller, observer and event-triggered matrices is established, and the stability of the scheme is analyzed. The results show that the proposed solution can effectively compensate DoS attacks and save network bandwidth resources by combining event-triggered mechanisms. Finally, a smart grid simulation example is employed to verify the feasibility and effectiveness of the scheme’s defense against DoS attacks.

## 1. Introduction

In recent years, with the development of computer networks and wireless communication technology, the rapid development of network control systems (NCSs) has led to a new round of industry change. With the emergence of 5G technology, more control systems can be combined with networks, and remote closed-loop NCSs can be formed through the network transmission of signals, which has been widely used in actual production [1,2,3]. Due to its wide range of applications, the stability and security of NCSs have attracted much attention in the academic community [4,5,6,7,8].

The combination of networks and control systems greatly improves the flexibility of all connected system devices. In other words, all system equipment can be connected through a wired or wireless network, replacing the original point-to-point control structure [9,10,11]. In [12,13,14], the authors analyzed the modeling and design problems of NCSs. NCSs mainly exchange control information through the network. Because the network is introduced during the control loop, a series of network problems (limited bandwidth resources, data dropout, etc.) are introduced into the control system, which greatly affect the performance of NCSs. In an open network environment, the security of control information is important. The most common network attack method of NCSs is a denial-of-service (DoS) attack. A DoS attack is a resource exhaustion-type attack. The primary purpose of a DoS attack is to render a computer or network incapable of providing normal services and to consume significant bandwidth resources. Ultimately, the lack of network bandwidth resources due to a DoS attack leads to serious consequences from such an attack, regardless of the processing speed of the computer, memory capacity or network bandwidth. For NCSs, DoS attacks prevent remote controllers and actuators from receiving feedback signals and control signals. Under DoS attacks, NCSs may be made unstable due to the lack of feedback measurement signals and control signals.

Based on the five issues of NCSs mentioned in [15] (sampled-data control, quantization control, networked control, event-triggered control and security control) and the analysis of NCSs exhibition trends and techniques, the security of NCSs can no longer be ignored. (i) For the current mainstream works on the safety of NCSs, NCSs mainly rely on digital sampling. The vast majority of DoS attacks can be modeled as packet loss of the control system. As a result, the security of NCSs primarily characterizes DoS attacks as packet loss. Currently, several design approaches for NCSs schemes have been reported in [16,17,18]. It is noteworthy that, except for a few works [19,20,21,22,23,24,25,26,27,28], most of the available results mentioned above do not consider the safety of NCSs but only discuss the design approach of NCSs schemes. For example, in [19], the stability of a network linear continuous-time system under power-constrained, known and unknown, pulse-width modulated DoS attack signals is investigated. In [20], a more general model for DoS attacks is proposed, where the characteristics of DoS attack are defined by the DoS attack frequency and the DoS attack duration. Based on similar ideas, extensions are made in [21] for dynamic output feedback controllers, in [22] for nonlinear networked control systems, and in [23] for distributed networked control systems. In [24], the stability and robustness of a network linear discrete-time system with random packet loss and random attacks is investigated. In [25], the zero-sum static game framework is applied to deal with the optimal control and scheduling problem of a linear network control system with communication constraints and DoS attacks. In [26], a state feedback resilient controller design method that relies on acyclically sampled data for the maximum and minimum durations of DoS attacks is presented by embedding a logic processor in the controller to capture DoS attack information and compute DoS parameters. Most of the results mentioned above are limited to performance analysis and do not explore controller synthesis [27]. Furthermore, these results assume the availability of system-wide state information, which limits their application in real control systems where only preliminary observations of the actual system state are measurable, usually due to limited communication and bandwidth resources or unavoidable noise [28]. (ii) In addition to the security issues caused by DoS attacks in NCSs, the network bandwidth resources are also very limited, reducing the frequent use of network bandwidth resources is also an urgent problem to be solved. The reduction of network bandwidth consumption by event-triggered control (ETC) has been extensively studied (see [16,29] for a single continuous-time system and [30,31] for multiagent systems), which makes ETC a promising solution for limited bandwidth resources of NCSs. Based on the above observations, is it possible to develop a unified observer-based output feedback scheme that can maintain system performance (stability) and compensate for the loss of control information caused by DoS attacks, while greatly saving limited network bandwidth resources and being more suitable for real-life scenarios (greater use of network convenience)? It is the focus of this paper to address this set of problems in a way that can be uniformly handled when the simultaneous effects of malicious DoS attacks, limited bandwidth resources, and unavailability of all state information are present.

**Figure 1:**
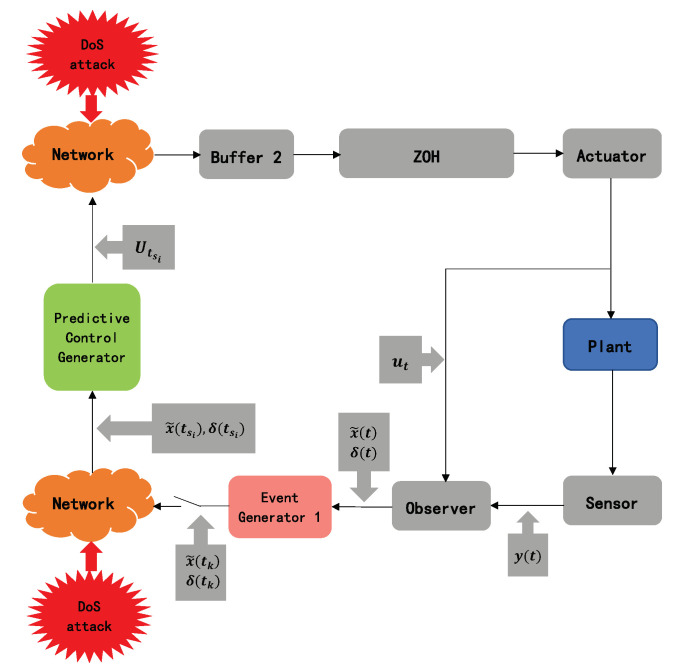
The diagram of the observer-based event-triggered predictive control (OB-ETPC) systems under denial-of-service (DoS) attacks.

This paper proposes a novel observer-based predictive control defense scheme, as shown in Figure 1, called observer-based event-triggered predictive control (OB-ETPC). Under this framework, we will study the observer-based predictive control system to actively compensate for data dropout due to a DoS attack and use the basic ideas of predictive control, linear matrix inequality (LMI) and Lyapunov function methods to solve the corresponding problems of event-triggered matrices, observer gain matrices and controller matrices. This work represents a significant expansion of previous results involving predictive control (PC) and the event-trigger mechanism (ET) under a DoS attack. The advantages of the proposed control defense scheme are fourfold:

(1) Our method is very different to that in the works of networked PCs [32,33,34] which have used time-triggered communication schemes. This paper adopts event-triggered predictive communication schemes to design a controller. Whether the observer’s state measurement information is sent depends on the error between the current observer state and the observer state of the most recently sent information. The event-triggered generator on the controller side greatly reduces the size of the sent predictive control sequences, greatly reduces the occupation of bandwidth resources and can also meet the needs of control performance [35].
(2) Compared with the existing predictive control compensation scheme for DoS attacks [7], another advantage of the OB-ETPC scheme adopted in this paper is the combination of the advantages of PC and ET [27,36,37,38,39,40,41,42]. With the combination of a model and static observer, it can cope with the problem that state information cannot be obtained directly and can also actively compensate for data packet dropout due to DoS attacks and greatly improve the stability of NCSs under DoS attacks.
(3) Compared with the latest DoS attack compensation scheme, the method in [27] only considers DoS attacks from the controller to the actuator side. In real-life scenarios, the attack from the sensor to controller side is often through a network link. In this paper, the novel OB-ETPC solves the problem of DoS attacks on both the sensor-to-controller and controller-to-actuator sides, which is more in line with real-life scenarios.
(4) The OB-ETPC is established to actively compensate for DoS attacks in NCSs. The observer gain matrix L and controller gain matrix K are co-designed based on the Lyapunov function method, and related criteria for event-triggered matrices are proposed based on linear matrix inequalities (LMIs).

The remainder of this paper is organized as follows. Section 2 deals with the problem descriptions and preliminaries. Section 3 considers OB-ETPC and the stability analysis of NCSs under DoS attacks. Section 4 verifies the feasibility of OB-ETPC under DoS attacks through a simulation example. We draw conclusions in Section 5.

All notations used in this paper are defined in the Table 1.

## 2. Problem Descriptions and Preliminaries

In this paper, we will study an observer-based state feedback networked control system under DoS attacks, as shown in Figure 1. The sensor component and the control component and the control component and the actuator component are connected through the network. Due to the openness and vulnerability of the network, NCSs are vulnerable to DoS attacks [43,44].

It is assumed that the dynamic evolution law of the controlled plant can be described by the following discrete system:

(1) $$\begin{array}{c} \begin{array}{cc} x(t+1) & =Ax(t)+Bu(t),\\ y(t) & =Cx(t). \end{array} \end{array}$$

where $x\left(t\right)\in R^{n}$ represents the state vector, $u\left(t\right)\in R^{m}$ represents the control vector and $y\left(t\right)\in R^{q}$ represents the device output vector. *A*, *B* and *C* are the appropriate dimension matrices of system (1) and *K* is the feedback gain matrix (to be solved below). The initial state of system (1) is $x\left(t_{0}\right)=x\left(0\right)$.

### Description of Each Component

(1) Sensor: The high-sensitivity sensor sends the output signal from plant to the observer [45].
(2) Observer: In reality, most systems cannot directly obtain the system’s state vector x(t). Using u(t)=Cx(t) to analyze the problem is restrictive and inaccurate. Therefore, in order to estimate plant state information, the observer is introduced into the NCSs. The full-dimension state observer is
  (2) $$\begin{array}{cc} \tilde{x}\left(t+1\right) & =A\tilde{x}\left(t\right)+Bu\left(t\right)+L\left(y\left(t\right)-\tilde{y}\left(t\right)\right),\\ \tilde{y}\left(t\right) & =C\tilde{x}\left(t\right). \end{array}$$
  where $\tilde{x}\left(t\right)\in R^{n}$ is the state vector of the observer, $\tilde{y}\left(t\right)\in R^{q}$ is the output vector of the observer and L is the gain matrix of the observer. We define δ(t) as the observer state error. Then,
  (3) $$\begin{array}{cc} \delta (t) & =x\left(t\right)-\tilde{x}\left(t\right),\\ \delta (t_{0}) & =x\left(t_{0}\right)-\tilde{x}\left(t_{0}\right), \end{array}$$
  and the observer error system can be described by
  (4) $$\begin{array}{cc} \delta (t+1) & =x\left(t+1\right)-\tilde{x}\left(t+1\right)\\ \; & =Ax\left(t\right)+Bu\left(t\right)-[A\tilde{x}\left(t\right)+Bu\left(t\right)+L\left(y\left(t\right)-\tilde{y}\left(t\right)\right]\\ \; & =(A-LC)\delta (t)\\ \; & ={\left(A-LC\right)}^{t+1}\delta \left(t_{0}\right). \end{array}$$
(3) Event Generator 1: Due to the limitation of network bandwidth resources, in order to reduce the transmission of data packets, prevent network congestion and improve the utilization of network bandwidth resources and the performance of NCSs, Event Generator 1 is designed on the sensor side to determine whether data packets need to be transmitted to the controller side [46].
  In this paper, we first introduce the event-triggered scheme in Event Generator 1 and assume that the time to trigger the Event Generator 1 is $t_{k}$(k=1,2,…,); then, the observer state information which is transmitted at this time is $\tilde{x}\left(t_{k}\right)$. The next trigger moment is
  (5) $$\begin{array}{ccc} t_{k+1} & = & t_{k}+\min\left\{r_{k},M\right\},\\ r_{k} & = & \min_{r}\left\{r|{\left[\tilde{x}\left(t_{k}+r\right)-\tilde{x}\left(t_{k}\right)\right]}^{T}\Phi \left[\tilde{x}\left(t_{k}+r\right)-\tilde{x}\left(t_{k}\right)\right]>\mu \tilde{x}{\left(t_{k}+r\right)}^{T}\Phi \tilde{x}\left(t_{k}+r\right)\right\}. \end{array}$$
  where μ>0 is a given scalar, M is a given positive integer, and Φ is a positive definite weight matrix. According to the above condition (5), the next trigger time is determined by the current observer state $\tilde{x}\left(t_{k}+r\right)$ and the observer state $\tilde{x}\left(t_{k}\right)$ at the latest trigger time, μ and Φ. Therefore, for μ>0 and Φ>0, if $f(t_{k}+r,t_{k})\leq 0$, the state data packets at $t_{k}+r$ need not be transmitted.
  (6) $$f\left(t_{k}+r,t_{k}\right)≜{\left[\tilde{x}\left(t_{k}+r\right)-\tilde{x}\left(t_{k}\right)\right]}^{T}\Phi \left[\tilde{x}\left(t_{k}+r\right)-\tilde{x}\left(t_{k}\right)\right]-\mu \tilde{x}{\left(t_{k}+r\right)}^{T}\Phi \tilde{x}\left(t_{k}+r\right).$$
  In other words, the embedded trigger condition of the Event Generator 1 is
  (7) $$f(t_{k}+r,t_{k})>0.$$
  When the trigger condition (7) is satisfied, the observer’s state information and state error are transmitted through the network and released to the controller.
  **Remark** **1.** Reducing network bandwidth consumption through event-driven control (ETC) has been extensively studied in [16,29,30,31]. Thus, ETC provides a very promising option to solve the bandwidth resource problem of NCSs under DoS attacks.
  **Remark** **2.** The data packet which is transmitted from the observer to the controller includes $\tilde{x}\left(t_{k}\right)$ and $\delta (t_{k})$.
  **Remark** **3.** M is the upper limit of the trigger time interval given by us to prevent long-term non-triggering from affecting the stability of the system.
(4) Predictive control generator: Combined with the model-based event-triggered predictive control (MB-ETPC) system, the plant’s predictive model is introduced on the control side. The predictive model is used to actively compensate a DoS attack and generate corresponding predictive control sequences. Then, Event Generator 2 is introduced at the control side, which is used to reduce the sending size of the predictive control sequences and further reduce the occupation of bandwidth resources. The predictive control sequences that trigger Event Generator 2 are packaged into a single data packet and sent to the actuator side through the network.
(5) Buffer: The buffers are used to store the incoming data packets.
(6) Zero-order holder (ZOH): The ZOH is used to choose a suitable control signal with a hold event interval of $\Omega =[t_{s_{i}},t_{s_{i+1}})$. $t_{s_{i}}$ is the moment that the predictive control generator successfully receives the data.
(7) Actuator: The function of the actuator is to receive the control signal from the ZOH and control the plant.
  In order to facilitate the analysis, we make the following assumptions regarding the above OB-ETPC system:
  **Assumption** **1.** *System (1) performs isochronous sampling. The sampling time is h, and all data packets are time-stamped.*
  **Assumption** **2.** *The sensor is time-driven, and the predictive controller and actuator are event-driven.*
  **Assumption** **3.** *This paper does not consider the time delay of the system and the delay of the transmission process.*
  **Assumption** **4.** *Assume that (A, B) is completely controllable and (A, C) is completely observable.*
  **Assumption** **5.** *$\tilde{x}\left(t_{0}\right)$ and $\delta (t_{0})$ are successfully sent at the initial moment $t_{0}$ from the observer to the controller.*

## 3. OB-ETPC of NCSs under DoS Attacks and Stability Analysis

### 3.1. DoS Attack Description

Denial-of-service (DoS) attacks are simple and effective attacks against a server. The purpose of DoS attacks is to allow the attacked host and server to deny normal users access and disrupt the normal operation of the system. Internet users cannot reach the attacked server and host, causing the server to fail [47]. DoS attacks occur on the sensor-to-controller and controller-to-actuator communication channels. Under DoS attacks, the network control systems will become unstable due to the lack of feedback measurement signals and control signals. Several typical examples of defenses against DoS attacks on modern NCSs are as follows: the United States specifically established the “National Infrastructure Protection Plan” in 2006 and the “Control System Security Plan (CSSP)” in 2010 to incorporate the protection of related national infrastructure control systems into national strategic plans, the European Union released the “European Program for Critical Infrastructure Protection (EPCIP)” in 2013 and the Ministry of Industry and Information Technology (MIIT) in China issued the “Notice on Strengthening the Information Security Management of Industrial Control Systems” in September 2011 [48].

**Figure 2:**
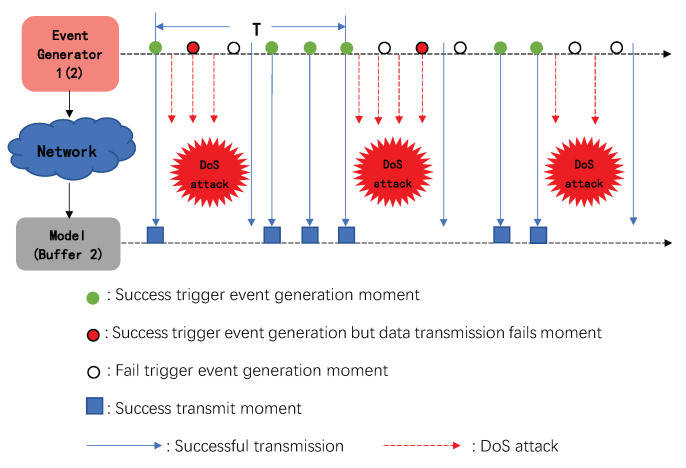
Diagram of information transmission under DoS attacks.

Due to DoS attacks affecting the communication channel, at this time, the observer state data packets released to the control end by Event Generator 1 and the system control sequence packets sent to the actuator side will suffer data dropout due to the DoS attacks. The information transmission under DoS attacks is shown in Figure 2.

**Figure 3:**
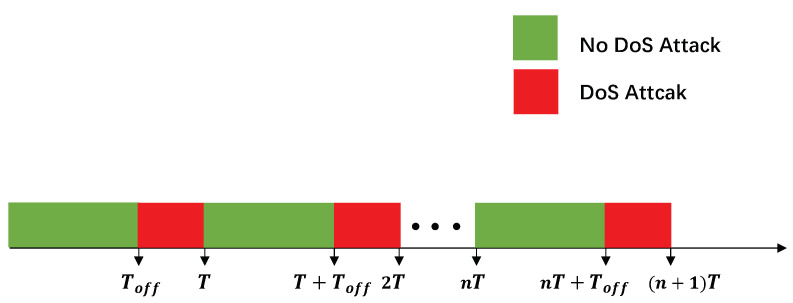
Diagram of the DoS attacks’ periodicity.

This paper considers periodic DoS attacks of a variable duration, which is a more general approach. According to [49], the model of DoS attacks is described as follows:

(8) $$I_{DoS}=\left\{\begin{array}{cc} 0 & ,t\in [nT,nT+T_{off}),\\ 1 & ,t\in [nT+T_{off},nT+T). \end{array}\right.$$

where n∈R represents the number of the DoS attack cycle, ${⋃}_{n\in R}\left[nT,nT+T_{off}\right)$ represents the time interval without DoS attacks and ${⋃}_{n\in R}\left[nT+T_{off},nT+T\right)$ represents the time interval of DoS attacks; see Figure 3.

The main aim of this paper is to use the idea of predictive control to actively compensate for the loss of triggered data packets due to DoS attacks. Based on the received observer state signal $\tilde{x}\left(t_{s_{i}}\right)$, the prediction controller not only needs to calculate the current control signal $u(t_{s_{i}})$ but also to perform continuous control serial prediction based on the current observer state signal. The generated data packets $U_{t_{s_{i}}}$ are stored in Buffer 2. The ZOH chooses the suitable control signal to control the plant, which plays an active role in compensating for data packet loss due to DoS attacks.

**Remark** **4.** *In the process of DoS attack compensation, the ZOH adopts a time-driven mechanism. The ZOH continuously sends the $u(t_{s_{i}})$ of Buffer 2 to the executor until a split-second before ${\hat{t}}_{s_{i+1}}$. If the packet in Buffer 2 is not updated before ${\hat{t}}_{s_{i+1}}$, the ZOH will send the data $\hat{u}\left({\hat{t}}_{s_{i+1}}|t_{s_{i}}\right)$ of the latest packet in Buffer 2 to the actuator continuously, and so on, until the packet of Buffer 2 is updated. If the packet in Buffer 2 is updated, the new packet is used to perform the above compensation mechanism.*

**Remark** **5.** *At present, several design methods for network NCSs schemes have been reported in [16,17,18] (except for a few works [19,20,21,22,24,25] ). Most of the above design schemes do not consider the security of network NCSs but only discuss the design methods of NCSs schemes. Therefore, exploring NCSs compensation under DoS attack is particularly important to solve the security problem of NCSs.*

**Assumption** **6.** *DoS attacks lead to a data packet dropout rate of 100%.*

**Assumption** **7.** *This paper considers periodic DoS attacks, and the period of DoS attacks is T. Because the duration of DoS attacks is variable, we define a real number $T_{off}^{min}\in \left(0,+\infty \right)$ which satisfies $T_{off}^{min}\leq T_{off}<T$ and $p≜T-T_{off}^{min}$.*

**Assumption** **8.** *The time interval between two adjacent DoS attacks is greater than M.*

### 3.2. OB-ETPC of NCSs under DoS Attacks

**Figure 4:**
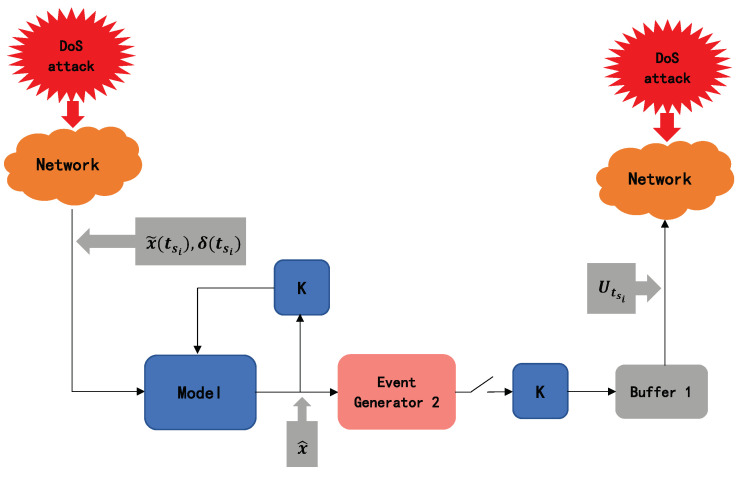
The block diagram of the predictive control generator.

Most of the currently researched DoS attack compensation predictive controllers directly compensate for data packet loss by predicting the dynamic evolution of NCSs under a predictive event-driven mechanism. In order to prevent DoS attacks, this paper uses a new predictive controller which combines ET and PC. We assume that the predictive model of the plant is known. The OB-ETPC actively compensates for DoS attacks to ensure system stability and ultimately achieve defense against DoS attacks. Therefore, the structure of the OB-ETPC controller design is shown in Figure 4.

Due to the existence of DoS attacks, the observer’s state information ${\left\{\tilde{x}\left(t_{k}\right)\right\}}_{k=1}^{\infty }$ triggered by Event Generator 1 cannot be completely transmitted because of data dropout. Thus, we introduce ${\left\{\tilde{x}\left(t_{s_{i}}\right)\right\}}_{i=1}^{\infty }$ to present the observer’s state information which is successfully received at times ${\left\{t_{s_{i}}\right\}}_{i=1}^{\infty }$; then, ${\left\{\tilde{x}\left(t_{s_{i}}\right)\right\}}_{i=1}^{\infty }\subseteq {\left\{\tilde{x}\left(t_{k}\right)\right\}}_{k=1}^{\infty }$.

**Remark** **6.** *Compared with the latest DoS attack compensation scheme [27], when network communication is introduced from the sensor to controller, the proposed OB-ETPC solves the DoS attack problem from the sensor to controller and controller to actuator.*

**Remark** **7.** *Based on Remark 2 and the existence of DoS attacks, we define ${\left\{\delta \left(t_{s_{i}}\right)\right\}}_{i=1}^{\infty }$ to present the observer’s state error which is successfully received at time ${\left\{t_{s_{i}}\right\}}_{i=1}^{\infty }$, then ${\left\{\delta \left(t_{s_{i}}\right)\right\}}_{i=1}^{\infty }\subseteq {\left\{\delta \left(t_{k}\right)\right\}}_{k=1}^{\infty }$.*

**Remark** **8.** *Based on Assumption 7, Assumption 8 and the event-triggering condition (5), the times $t_{s_{i+1}}$ and $t_{s_{i}}$ of two successful transmissions satisfy the following relationship: $t_{s_{i+1}}-t_{s_{i}}\leq 2\times M+p$.*

The predictive model system is

(9) $$\hat{x}\left(t+1\right)=A\hat{x}\left(t\right)+Bu\left(t\right)+LC\delta \left(t\right).$$

Next, we will explain in detail the use of OB-ETPC to prevent DoS attacks and actively compensate for state data packet loss due to DoS attacks. Due to the impact of DoS attacks, the moment of the successfully received from observer is $t_{s_{i}}$. As long as the observer’s state $\tilde{x}\left(t_{s_{i}}\right)$ is successfully received by the predictive controller, it will be predicted by the predictive model system (9). The predictive model will perform prediction to obtain the corresponding predictive control sequences and actively compensate for the DoS attack. The closed-loop state prediction at the future trigger moment is as follow:

(10) $$\begin{array}{ccc} \hat{x}\left(t_{s_{i}}|t_{s_{i}}\right) & = & \tilde{x}\left(t_{s_{i}}\right), \end{array}$$

(11) $$\begin{array}{ccc} \delta (t_{s_{i}}|t_{s_{i}}) & = & \delta (t_{s_{i}}), \end{array}$$

(12) $$\begin{array}{ccc} \hat{x}\left(t_{s_{i}}+1|t_{s_{i}}\right) & = & A\hat{x}\left(t_{s_{i}}|t_{s_{i}}\right)+BK\hat{x}\left(t_{s_{i}}|t_{s_{i}}\right)+LC\delta \left(t_{s_{i}}|t_{s_{i}}\right), \end{array}$$

(13) $$\begin{array}{ccc} \hat{x}\left(t_{s_{i}}+j+1|t_{s_{i}}\right) & = & A\hat{x}\left(t_{s_{i}}+j|t_{s_{i}}\right)+BKx\left(t_{s_{i}}+j|t_{s_{i}}\right)+LC\delta \left(t_{s_{i}}+j|t_{s_{i}}\right), \end{array}$$

(14) $$\begin{array}{ccc} j & = & 1,2,\ldots ,{\hat{t}}_{s_{i+1}}-t_{s_{i}}-1, \end{array}$$

(15) $$\begin{array}{ccc} & \vdots & \end{array}$$

(16) $$\begin{array}{ccc} \hat{x}\left({\hat{t}}_{s_{i+m}}+j+1|t_{s_{i}}\right) & = & A\hat{x}\left({\hat{t}}_{s_{i+m}}+j|t_{s_{i}}\right)+BK\hat{x}\left({\hat{t}}_{s_{i+m}}+j|t_{s_{i}}\right)+LC\delta \left({\hat{t}}_{s_{i+m}}+j|t_{s_{i}}\right), \end{array}$$

(17) $$\begin{array}{ccc} j & \in & \left\{0,1,2,\ldots ,{\hat{t}}_{s_{i+m+1}}-{\hat{t}}_{s_{i+m}}-1\right\}, \end{array}$$

(18) $$\begin{array}{ccc} m & \in & \left\{1,2,\ldots ,l_{i}\right\}, \end{array}$$

where $\delta \left(t_{s_{i}}+j|t_{s_{i}}\right)={\left(A-LC\right)}^{j}\delta \left(t_{s_{i}}\right)$ and $\delta \left({\hat{t}}_{s_{i+m}}+j|t_{s_{i}}\right)={\left(A-LC\right)}^{j}\delta \left({\hat{t}}_{s_{i+m}}|t_{s_{i}}\right)$.

In order to reduce the size of the predictive control sequences that need to be sent, Event Generator 2 is introduced into the predictive control generator. ${\hat{t}}_{s_{i+1}}$ is the first predictive event-triggered moment:

(19) $$\begin{array}{ccc} {\hat{t}}_{s_{i+1}} & = & t_{s_{i}}+\min\left\{r_{s_{i}},M\right\},\\ r_{s_{i}} & = & \min_{r}\left\{r|{\left[\hat{x}\left(t_{s_{i}}+r|t_{s_{i}}\right)-\hat{x}\left(t_{s_{i}}|t_{s_{i}}\right)\right]}^{T}\Phi \left[\hat{x}\left(t_{s_{i}}+r|t_{s_{i}}\right)-\hat{x}\left(t_{s_{i}}|t_{s_{i}}\right)\right]\right.\\ & & \left.>\mu \hat{x}{\left(t_{s_{i}}+r|t_{s_{i}}\right)}^{T}\Phi \hat{x}\left(t_{s_{i}}+r|t_{s_{i}}\right)\right\}, \end{array}$$

and

(20) $$\begin{array}{ccc} {\hat{t}}_{s_{i+m+1}} & = & {\hat{t}}_{s_{i+m}}+\min\left\{r_{s_{i+m}},M\right\},\\ r_{s_{i+m}} & = & \min_{r}\left\{r|{\left[\hat{x}\left({\hat{t}}_{s_{i+m}}+r|t_{s_{i}}\right)-\hat{x}\left({\hat{t}}_{s_{i+m}}|t_{s_{i}}\right)\right]}^{T}\Phi \left[\hat{x}\left({\hat{t}}_{s_{i+m}}+r|t_{s_{i}}\right)-\hat{x}\left({\hat{t}}_{s_{i+m}}|t_{s_{i}}\right)\right]\right.\\ & & \left.>\mu \hat{x}{\left({\hat{t}}_{s_{i+m}}+r|t_{s_{i}}\right)}^{T}\Phi \hat{x}\left({\hat{t}}_{s_{i+m}}+r|t_{s_{i}}\right)\right\},\\ m & \in & \left\{1,2,\ldots ,l_{i}\right\}, \end{array}$$

where μ and Φ are given in condition (5). Therefore, the predicted moment of transmission is $T_{s_{i}}=\left\{\begin{array}{cccc} {\hat{t}}_{s_{i+1}}, & {\hat{t}}_{s_{i+2}}, & \ldots , & {\hat{t}}_{s_{i+l_{i}}} \end{array}\right\}.$

**Remark** **9.** *Based on Remarks 3,8 and Assumptions 7−8, $l_{i}$ satisfies ${\hat{t}}_{s_{i+l_{i}}}\leq t_{s_{i}}+2\times M+p<{\hat{t}}_{s_{i+l_{i}+1}}.$*

Then, $l_{i}$ predictive event-triggered states are packed into $\hat{X}\left(t_{s_{i}}\right)$.

$$\hat{X}\left(t_{s_{i}}\right)=\left[\begin{array}{c} \tilde{x}\left(t_{s_{i}}\right),\hat{x}\left({\hat{t}}_{s_{i+1}}|t_{s_{i}}\right),\cdots ,\hat{x}\left({\hat{t}}_{s_{i+l_{i}}}|t_{s_{i}}\right) \end{array}\right].$$

In order to fully respond to DoS attacks, the controller generates $l_{i}$ predictive control sequences based on the predictive event-triggered states $\hat{X}\left(t_{s_{i}}\right)$.

According to the corresponding predictive controller law u(t)=Kx(t), the controller’s predictive control can be obtained:

(21) $$\begin{array}{ccc} u(t_{s_{i}}) & = & K\tilde{x}\left(t_{s_{i}}\right), \end{array}$$

(22) $$\begin{array}{ccc} \hat{u}\left({\hat{t}}_{s_{i+1}}|t_{s_{i}}\right) & = & K\hat{x}\left({\hat{t}}_{s_{i+1}}|t_{s_{i}}\right), \end{array}$$

(23) $$\begin{array}{ccc} & \vdots & \end{array}$$

(24) $$\begin{array}{ccc} \hat{u}\left({\hat{t}}_{s_{i+l_{i}}}|t_{s_{i}}\right) & = & K\hat{x}\left({\hat{t}}_{s_{i+l_{i}}}|t_{s_{i}}\right). \end{array}$$

Then control sequences are generated as:

$$U_{t_{s_{i}}}≜\left[\begin{array}{ccccc} u(t_{s_{i}}), & \hat{u}\left({\hat{t}}_{s_{i+1}}|t_{s_{i}}\right), & \hat{u}\left({\hat{t}}_{s_{i+2}}|t_{s_{i}}\right), & \cdots , & \hat{u}\left({\hat{t}}_{s_{i+l_{i}}}|t_{s_{i}}\right) \end{array}\right].$$

The generated $l_{i}$ predictive control signals are stored in Buffer 2 for the ZOH to select a suitable control input signal. Then, the ZOH sends the selected control input signal to the actuator to complete the defense against the DoS attack.

Compared with other PC approaches [32] without Event Generator 2, all predicted control sequences will be packed, and the predicted control sequences to be sent are ${\hat{U}}_{t_{s_{i}}}=\left[u\left(t_{s_{i}}\right),\hat{u}\left(t_{s_{i}}+1|t_{s_{i}}\right),\hat{u}\left(t_{s_{i}}+2|t_{s_{i}}\right),\ldots ,\hat{u}\left(t_{s_{i}}+2\times M+p|t_{s_{i}}\right)\right].$ Obviously, after Event Generator 2 is added, the size of the predictive control sequences to be transmitted is greatly reduced, and the occupation of bandwidth resources is reduced.

### 3.3. The Closed-Loop System

**Lemma** **1.** *Comparing the observer system (2) and predictive model system (9), it is necessary to provide the proof for the following relationships.*

(25) $$\begin{array}{ccc} t_{s_{i+h}} & = & {\hat{t}}_{s_{i+h}}, \end{array}$$

(26) $$\begin{array}{ccc} \hat{x}\left({\hat{t}}_{s_{i+h}}\right) & = & \tilde{x}\left(t_{s_{i+h}}\right), \end{array}$$

(27) $$\begin{array}{ccc} \hat{x}\left(t\right) & = & \tilde{x}\left(t\right), \end{array}$$

(28) $$\begin{array}{ccc} h & = & 1,2,\ldots ,s_{i+1}-s_{i}-1, \end{array}$$

(29) $$\begin{array}{ccc} i & = & 1,2,\ldots ,\infty , \end{array}$$

(30) $$\begin{array}{ccc} t & \in & \left[t_{0},\infty \right)\cap \left[t_{s_{i}},t_{s_{i+1}}\right). \end{array}$$

According to Remark 7 and Lemma 1, the closed-loop system with DoS attack compensation under event-triggering condition (5) is expressed as

(31) $$\begin{array}{ccc} x(t+1) & = & Ax\left(t\right)+BK\hat{x}\left({\hat{t}}_{s_{i+m}}|t_{s_{i}}\right), \end{array}$$

(32) $$\begin{array}{ccc} \tilde{x}\left(t+1\right) & = & A\tilde{x}\left(t\right)+BK\hat{x}\left({\hat{t}}_{s_{i+m}}|t_{s_{i}}\right)+LC\delta \left({\hat{t}}_{s_{i+m}}|t_{s_{i}}\right), \end{array}$$

(33) $$\begin{array}{ccc} \hat{x}\left(t+1\right) & = & A\hat{x}\left(t\right)+BK\hat{x}\left({\hat{t}}_{s_{i+m}}|t_{s_{i}}\right)+LC\delta \left({\hat{t}}_{s_{i+m}}|t_{s_{i}}\right), \end{array}$$

(34) $$\begin{array}{ccc} \delta (t+1) & = & (A-LC)\delta (t), \end{array}$$

(35) $$\begin{array}{ccc} \hat{x}\left(t_{s_{i}}\right) & = & \tilde{x}\left(t_{s_{i}}\right), \end{array}$$

(36) $$\begin{array}{ccc} t & \in & \left[t_{s_{i}},t_{s_{i+1}}\right)\cap \left[{\hat{t}}_{s_{i+m}},{\hat{t}}_{s_{i+m+1}}\right), \end{array}$$

(37) $$\begin{array}{ccc} m & \in & \left\{1,2,\ldots ,l_{i}\right\}. \end{array}$$

Comparing the observer system (32) and predictive model system (33) in the closed-loop system, and to facilitate system analysis and controller design, for $t\in \left[t_{s_{i}},t_{s_{i+1}}\right)\cap \left[{\hat{t}}_{s_{i+m}},{\hat{t}}_{s_{i+m+1}}\right)$, we define $e_{s_{i}}\left(t\right)=\tilde{x}\left(t\right)-\tilde{x}\left(t_{s_{i+m}}\right)$. According to $e_{s_{i}}$ and δ(t), the above closed-loop system (31)–(37) can be written as

(38) $$\begin{array}{ccc} x(t+1) & = & Ax\left(t\right)+BKx\left(t\right)-BKe_{s_{i}}\left(t\right)-BK\delta \left(t\right), \end{array}$$

(39) $$\begin{array}{ccc} \delta (t+1) & = & (A-LC)\delta (t), \end{array}$$

(40) $$\begin{array}{ccc} t & \in & \left[t_{s_{i}},t_{s_{i+1}}\right)\cap \left[{\hat{t}}_{s_{i+m}},{\hat{t}}_{s_{i+m+1}}\right), \end{array}$$

(41) $$\begin{array}{ccc} m & \in & \left\{1,2,\ldots ,l_{i}\right\}. \end{array}$$

**Remark** **10.** *Note that $t_{s_{i+1}}=t_{s_{i}}+t_{s_{i+1}}-t_{s_{i}}\leq t_{s_{i}}+2\times M+p<{\hat{t}}_{s_{i+l_{i}+1}},$ and so the compensation selected from Buffer 2 by the ZOH is used to compensate for DoS attacks when $t\in [t_{s_{i}},t_{s_{i+1}})$.*

**Remark** **11.** *Comparing event-triggered conditions (5) and (20), they are found to be consistent. Based on Remark 9, no event is triggered when $t\in \left[t_{s_{i}},t_{s_{i+1}}\right)\cap \left[{\hat{t}}_{s_{i+m}},{\hat{t}}_{s_{i+m+1}}\right)$[41]. Thus, based on event-triggering condition (5) and $e_{s_{i}}$, the following inequality needs to be followed:*

(42) $$\begin{array}{c} \begin{array}{cc} e_{s_{i}}^{T}\left(t\right)\Phi e_{s_{i}}\left(t\right) & \leq \mu {\tilde{x}}^{T}\left(t\right)\Phi \tilde{x}\left(t\right),\\ t\in [t_{s_{i}},t_{s_{i+1}}) & \cap [{\hat{t}}_{s_{i+m}},{\hat{t}}_{s_{i+m+1}}). \end{array} \end{array}$$

### 3.4. Stability Analysis

In this subsection, the design method of the state feedback controller gain matrix *K*, observer gain matrix *L* and triggering parameter Φ in condition (5) will be given.

**Theorem** **1.** *Based on Assumptions 1–7, for given parameters 0<μ<1, the system (38)–(41) will be asymptotically stable if there are matrices $\tilde{P}>0$, $\tilde{\Phi }>0$, Q>0 and matrices X, G with appropriate dimensions such that the following LMI is satisfied:*

(43) $$\left[\begin{array}{cccccc} -\tilde{P} & * & * & * & * & *\\ 0 & -\tilde{\Phi } & * & * & * & *\\ 0 & 0 & -Q & * & * & *\\ A\tilde{P}+BX & -BX & -BX & -\tilde{P} & * & *\\ \mu \tilde{\Phi } & -\mu \tilde{\Phi } & -\mu \tilde{\Phi } & 0 & -\mu \tilde{\Phi } & *\\ 0 & 0 & QA-GC & 0 & 0 & -Q \end{array}\right]<0,$$

*with*

$$\Phi ={\tilde{P}}^{-1}\tilde{\Phi }{\tilde{P}}^{-1},K=X{\tilde{P}}^{-1},L=Q^{-1}G.$$

**Proof.**  To connect x(t) and δ(t), we choose an appropriate Lyapunov function as

$$V\left(x\left(t\right),\delta \left(t\right)\right)=x^{T}\left(t\right)Px\left(t\right)+\delta^{T}Q\delta \left(t\right),$$

where *P* and *Q* are symmetric positive definite matrices. When $t\in \left[t_{s_{i}},t_{s_{i+1}}\right)\cap \left[{\hat{t}}_{s_{i+m}},{\hat{t}}_{s_{i+m+1}}\right)$, calculating the difference of V(x(t),δ(t)) along the system (38)–(41) and taking the inequalities in condition (42) into account yields that

$$\begin{array}{ccc} & & \Delta V(x(t),\delta (t))\\ & = & V(x(t+1),\delta (t+1))-V(x(t),\delta (t))\\ & \leq & \left[x^{T}\left(t+1\right)Px\left(t+1\right)+\delta^{T}\left(t+1\right)Q\delta \left(t+1\right)\right]-\left[x^{T}\left(t\right)Px\left(t\right)+\delta^{T}\left(t\right)Q\delta \left(t\right)\right]\\ & & -e_{s_{i}}^{T}\left(t\right)\Phi e_{s_{i}}\left(t\right)+\mu {\tilde{x}}^{T}\left(t_{s_{i+m}}\right)\Phi \tilde{x}\left(t_{s_{i+m}}\right)\\ & = & {\left[Ax\left(t\right)+BKx\left(t\right)-BKe_{s_{i}}\left(t\right)-BK\delta \left(t\right)\right]}^{T}P\left[Ax\left(t\right)+BKx\left(t\right)-BKe_{s_{i}}\left(t\right)-BK\delta \left(t\right)\right]-x^{T}\left(t\right)Px\left(t\right)\\ & & -\delta^{T}\left(t\right)Q\delta \left(t\right)-e_{s_{i}}^{T}\left(t\right)\Phi e_{s_{i}}\left(t\right)+{\left[\left(A-LC\right)\delta \left(t\right)\right]}^{T}Q\left[\left(A-LC\right)\delta \left(t\right)\right]\\ & & +\mu {\left[x\left(t\right)-e_{s_{i}}-\delta \left(t\right)\right]}^{T}\Phi \left[x\left(t\right)-e_{s_{i}}-\delta \left(t\right)\right]\\ & = & \left[\begin{array}{ccc} x^{T}\left(t\right) & e_{s_{i}}^{T}\left(t\right) & \delta^{T}\left(t\right) \end{array}\right][\left[\begin{array}{ccc} -P & * & *\\ 0 & -\Phi & *\\ 0 & 0 & -Q \end{array}\right]+\left[\begin{array}{c} {\left(A+BK\right)}^{T}\\ {\left(-BK\right)}^{T}\\ {\left(-BK\right)}^{T} \end{array}\right]P\left[\begin{array}{ccc} \left(A+BK\right) & \left(-BK\right) & \left(-BK\right) \end{array}\right]\\ & & +\left[\begin{array}{c} 0\\ 0\\ {\left(A-LC\right)}^{T} \end{array}\right]Q\left[\begin{array}{ccc} 0 & 0 & \left(A-LC\right) \end{array}\right]+\mu \left[\begin{array}{c} I\\ -I\\ -I \end{array}\right]\Phi \left[\begin{array}{ccc} I & -I & -I \end{array}\right]]\left[\begin{array}{c} x(t)\\ e_{s_{i}}\left(t\right)\\ \delta (t) \end{array}\right]. \end{array}$$

By using Schur’s complement, if the following inequality is satisfied, ΔV(x(t),δ(t))<0 can be concluded.

(44) $$\left[\begin{array}{cccccc} -P & * & * & * & * & *\\ 0 & -\Phi & * & * & * & *\\ 0 & 0 & -Q & * & * & *\\ P(A+BK) & P(-BK) & P(-BK) & -P & * & *\\ \mu \Phi & -\mu \Phi & -\mu \Phi & 0 & -\mu \Phi & *\\ 0 & 0 & Q(A-LC) & 0 & 0 & -Q \end{array}\right]<0.$$

However, the above inequality is not in the form of LMI. To reduce it to a linear matrix inequality, we perform pre and post-multiplying (44) with

$$diag\{\begin{array}{cccccc} P^{-1}, & P^{-1}, & I, & P^{-1}, & P^{-1}, & I \end{array}\}.$$

The above matrix inequality is transformed into the following linear matrix inequality form:

(45) $$\left[\begin{array}{cccccc} -P^{-1} & * & * & * & * & *\\ 0 & -P^{-1}\Phi P^{-1} & * & * & * & *\\ 0 & 0 & -Q & * & * & *\\ \left(A+BK\right)P^{-1} & \left(-BK\right)P^{-1} & \left(-BK\right)P^{-1} & -P^{-1} & * & *\\ P^{-1}\mu \Phi P^{-1} & -P^{-1}\mu \Phi P^{-1} & -P^{-1}\mu \Phi P^{-1} & 0 & -P^{-1}\mu \Phi P^{-1} & *\\ 0 & 0 & Q(A-LC) & 0 & 0 & -Q \end{array}\right]<0.$$

We define $\tilde{P}≜P^{-1}$, $\tilde{\Phi }≜P^{-1}\Phi P^{-1}$, $X≜KP^{-1}$, G≜QL. According to the Lyapunov stability theory, we find that if the LMI in (43) holds, then the closed-loop system (38)–(41) is asymptotically stable. This completes the proof.  □

**Remark** **12.** *The main purpose of this paper is to apply the OB-ETPC to compensate for DoS attacks. Unlike the work in [50], this paper introduces two event-triggered generators that compensate for the DoS attack while saving a large amount of bandwidth resources.*

## 4. Simulation Example

In this section, we apply observer-based event-triggered predictive control to the smart grid example with a four-bus model of the distribution test feeders under DoS attacks [50]. The relevant parameters of the system can be found in [50,51]. The sampling time of the system is h=0.02s, the DoS attack cycle is T=2s and the trigger parameter is μ=0.08.

(46) $$A=\left[\begin{array}{cccc} 1.0156 & 0.0139 & 0.0457 & 0.0971\\ -0.0353 & 0.9997 & -0.0008 & -0.0017\\ -0.0526 & -0.0448 & 0.9625 & -0.0797\\ -0.0080 & -0.0505 & 0.0903 & 0.9011 \end{array}\right],\;B=\left[\begin{array}{cccc} -0.0025 & 0.0315 & 0.0514 & -0.1118\\ -0.0350 & 0.0006 & -0.0009 & 0.0019\\ -0.0042 & -0.0057 & -0.0422 & -0.0742\\ -0.0400 & -0.0392 & -0.0086 & -0.0980 \end{array}\right].$$

Other details of the system’s parameters can be found in [50]. Then, the matrix *C* is chosen as

(47) $$C=\left[\begin{array}{cccc} 1.0000 & 2.0000 & 0.0000 & 0.5000 \end{array}\right].$$

According to Theorem 1, using MATLAB to solve the corresponding linear matrix inequality (LMI), the corresponding controller gain matrix *K*, observer gain matrix *L* and event-triggering matrix Φ are obtained as follows:

(48) $$\begin{array}{ccc} K & = & \left[\begin{array}{cccc} 4.4570 & 9.2776 & -1.8638 & 1.0980\\ -4.6407 & -6.9638 & -1.0600 & 2.4232\\ 1.0259 & -0.2534 & 2.1682 & -1.2877\\ 0.3916 & -0.9666 & 0.9551 & 0.3309 \end{array}\right], \end{array}$$

(49) $$\begin{array}{ccc} L & = & \left[\begin{array}{c} -0.3809\\ 0.6965\\ 0.2680\\ 0.0721 \end{array}\right], \end{array}$$

(50) $$\begin{array}{ccc} \Phi & = & \left[\begin{array}{cccc} 0.2233 & 0.0067 & 0.1629 & 0.0964\\ 0.0067 & 0.2842 & -0.1596 & -0.0537\\ 0.1629 & -0.1596 & 0.2761 & 0.0572\\ 0.0964 & -0.0537 & 0.0572 & 0.2232 \end{array}\right]. \end{array}$$

The effectiveness of OB-ETPC in the defense of DoS attacks is demonstrated by comparing the experimental results of three simulation cases under DoS attacks with different durations and two other mainstream DoS attack compensation schemes based on time-triggered predictive control (TTPC) [52,53,54] and event-triggered control (ETC) [27,48,49]. Assume that the plant initial state is $x_{0}={\left[\begin{array}{cccc} 5.2 & 5.2 & 0.6 & 9.7 \end{array}\right]}^{T}$ and the observer initial state is ${\tilde{x}}_{0}={\left[\begin{array}{cccc} 5 & 4 & 0.4 & 6.4 \end{array}\right]}^{T}$, so the observer state initial error is $\delta_{0}={\left[\begin{array}{cccc} 0.2 & 1.2 & 0.2 & 3.3 \end{array}\right]}^{T}$.

**Figure 5:**
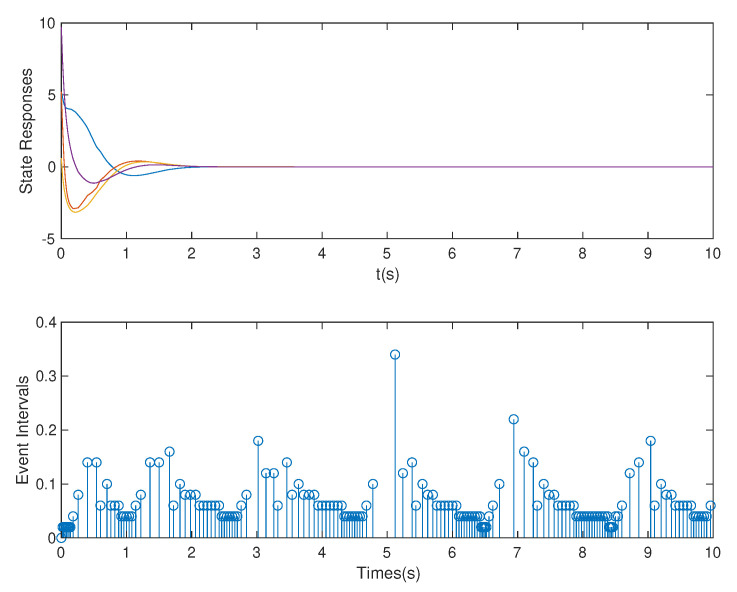
State responses and event intervals with DoS attack for OB-ETPC.

**Figure 6:**
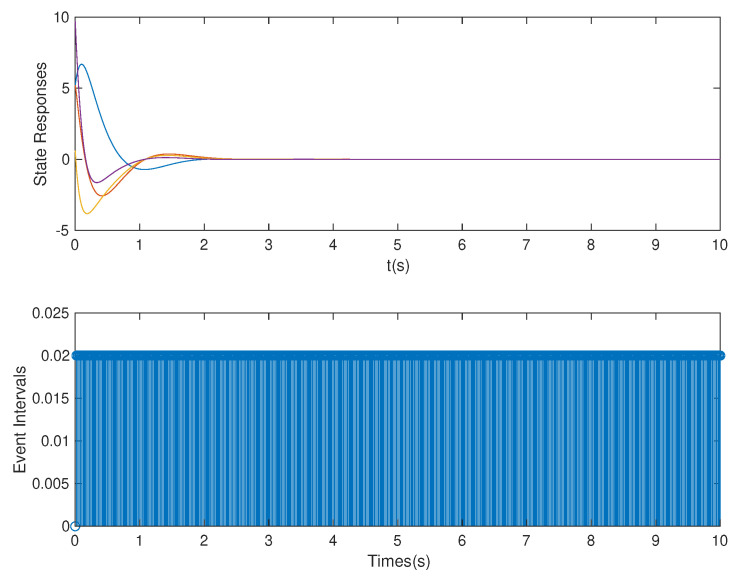
State responses and event intervals with DoS attacks for time-triggered predictive control (TTPC).

**Case** **1.** *In this case, based on the controller gain matrix K and observer gain matrix L obtained by the above OB-ETPC and based on TTPC [52,53,54], the state responses and event intervals of the system with DoS attack are shown in Figure 5 and Figure 6, respectively.*

*As shown in Figure 5 and Figure 6, we can clearly see that both OB-ETPC-based or TTPC-based methods are able to make the system state stable when encountering DoS attacks. In this case, based on the OB-ETPC and the TTPC, all packet losses due to DoS attacks (p=1* s *or p=1.5* s*) are fully compensated. From Table 2 we can see that, based on OB-ETPC, there are 154 triggered moments within 500 sampling times, and the average triggered time interval is 0.0649 s. Based on the TTPC, the system has 500 triggered moments when compensating for DoS attacks, and the average triggered time interval is 0.02* s. *Moreover, it is also clear from the data in the Table 2 that the amount of data packets required to stabilize the OB-ETPC-based system is 114, while the amount of data packets required to stabilize the TTPC-based system is up to 380 when encountering weak DoS attacks. When encountering strong DoS attacks, the OB-ETPC-based system requires 54 data packets for stability, while the TTPC-based system requires 130 data packets for stability. Thus, the event-triggered mechanism not only does not degrade the performance of the system, but also greatly reduces the network bandwidth resource consumption.*

**Figure 7:**
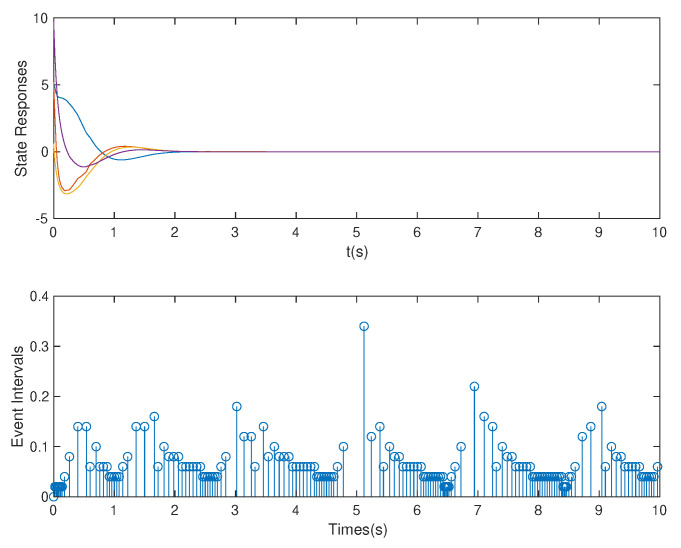
State responses and event intervals with DoS attacks (p=1 s) for OB-ETPC.

**Figure 8:**
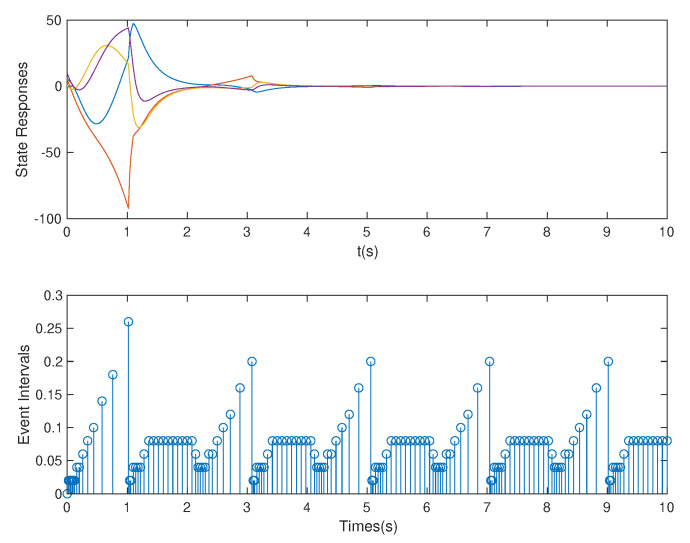
State responses and event intervals with DoS attacks (p=1 s) for ETC.

**Case** **2.** *In this case, we assume that p=1* s *is used. Based on the controller gain matrix K and observer gain matrix L obtained by the above OB-ETPC and based on the ETC [27,48,49], the state responses and event intervals of the system are shown in Figure 7 and Figure 8, respectively.*

*As shown in Figure 7 and Figure 8, the system can be seen to experience a weak DoS attack. From Table 2 we can see that, based on OB-ETPC, there are 154 triggered moments and the average triggered time interval is 0.0649* s. *In this case, based on ETC, there are 143 triggered moments and the average triggered time interval is 0.0699* s. *Because the simulation case is performed in the upper bound of the weak DoS attack, according to Assumption 7, the ETC-based method can defend against DoS attacks with an arbitrary duration in the weak attack duration range and remain stable after a period of time.*

**Figure 9:**
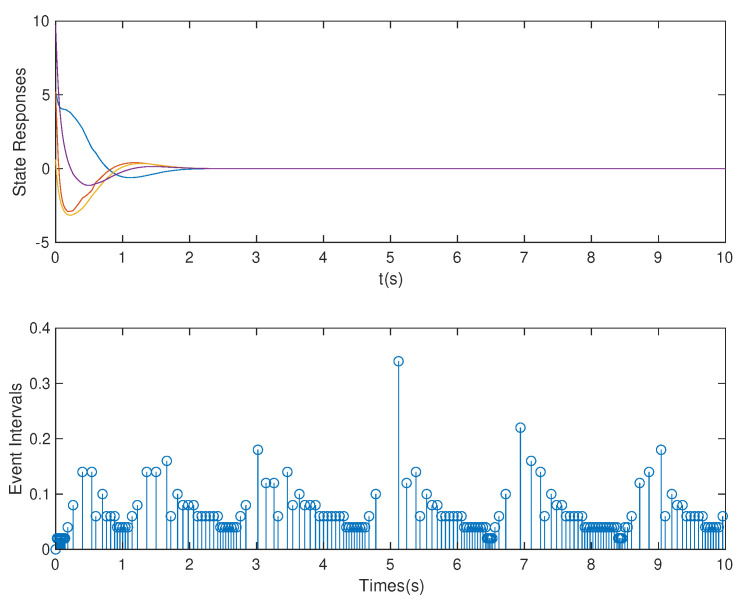
State responses and event intervals with DoS attacks (p=1.5 s) for OB-ETPC.

**Figure 10:**
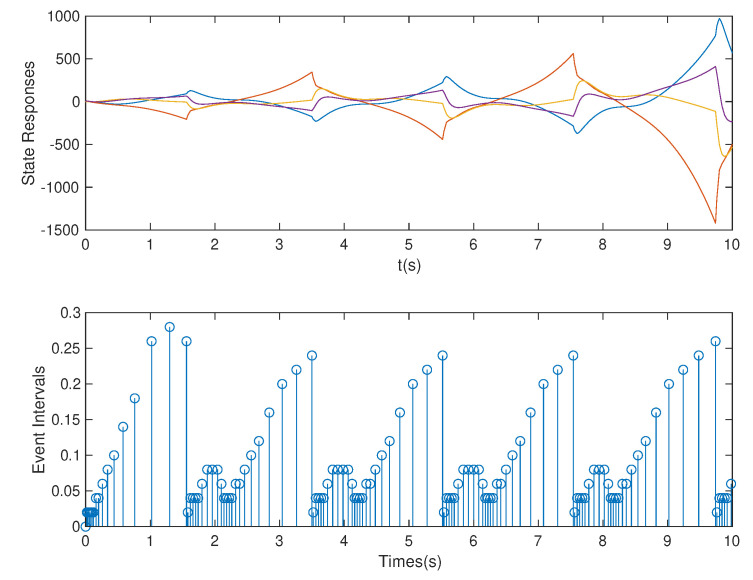
State responses and event intervals with DoS attacks (p=1.5 s) for ETC.

**Case** **3.** *In this case, we assume that p=1.5* s *is used. Based on the controller gain matrix K and observer gain matrix L obtained by the above OB-ETPC and based on the ETC [27,48,49], the state responses and event intervals of the system are shown in Figure 9 and Figure 10, respectively.*

*As shown in Figure 9 and Figure 10, the system can be seen to experience strong DoS attacks. From Table 2, we can see that, based on OB-ETPC, there are 154 triggered moments and the average triggered time interval is 0.0649* s. *In this case, based on ETC, there are 117 triggered moments and the average triggered time interval is 0.0855* s. *In this case, the ETC-based method cannot defend against strong DoS attacks and the system loses stability. However, since OB-ETPC can fully compensate for DoS attacks, the system remains stable after encountering strong DoS attacks.*

**Remark** **13.** *The data numbers represent the amount of data successfully transmitted to the actuator.*

Compared with TTPC [52,53,54], the above results verify the effectiveness of our proposed OB-ETPC in reducing bandwidth resource consumption. Furthermore, compared to ETC [27,48,49], the above results verify the feasibility of our proposed OB-ETPC approach to defend against DoS attacks. In summary, the OB-ETPC approach proposed in this paper can make up for the deficiency of different types of NCS compensation schemes under DoS attacks. In the case that the system state cannot be completely measured, OB-ETPC can ensure the stability of NCSs and reduce the occupancy of bandwidth resources, which cannot be achieved by other methods at present.

## 5. Conclusions

This paper studies the problem of event-triggered control based on a static observer in networked control systems (NCSs) under DoS attacks. The OB-ETPC is a new method to solve the problem of DoS attacks. The results show that the introduction of an observer and predictive model in the system has a significant effect on the defense against DoS attacks. The establishment of an event-triggered scheme greatly reduces the size of the predictive control sequence compensation packet. In addition, a ZOH is constructed in the actuator node to actively compensate for data packet loss due to DoS attacks. The OB-ETPC is an active compensation method for NCSs under DoS attacks that combines event-triggered conditions, robust controller-feedback gain and observer gain. The practical application example shows that this method can not only actively compensate for DoS attacks but can also reduce the bandwidth occupancy while maintaining the stability of the NCSs.

In future research, it would be beneficial to extend the OB-ETPC to the defense against DoS attack of distributed NCSs. In distributed NCSs, it is necessary to consider the impact of network-induced delay, data packet dropout and DoS attacks on the closed-loop system. In addition, the OB-ETPC approach would also be of great significance for solving noise problems [55].

## Figures and Tables

**Table 1:** All notations in this paper.

Notations	Definitions
$x\left(t\right)\in R^{n}$	The state vector.
$u\left(t\right)\in R^{m}$	The control vector.
$y\left(t\right)\in R^{q}$	The device output vector.
$\tilde{x}\left(t\right)\in R^{n}$	The state vector of the observer.
$\tilde{y}\left(t\right)\in R^{q}$	The output vector of the observer.
$\hat{x}\left(t\right)\in R^{n}$	The state vector of the predictive control generator.
δ(t)	The observer state error.
$t_{k}$ (k=1,2,…,)	The time to trigger the Event Generator 1.
$t_{s_{i}}$	The moment at which the predictive control generator that successfully receives the data.
*A*, *B* and *C*	The appropriate dimension matrices of the system.
*L*	The gain matrix of the observer.
*K*	The feedback gain matrix.
μ>0	A given scalar.
*M*	A given positive integer.
Φ	A positive definite weight matrix.
*T*	The period of DoS attacks.
n∈R	The number of the DoS attack cycle.
$T_{off}^{min}\in \left(0,+\infty \right)$	A real number which satisfies $T_{off}^{min}\leq T_{off}<T$.
*p*	A real number which satisfies $p≜T-T_{off}^{min}$.
*M*	A given positive integer.
*P* and *Q*	The symmetrical positive definite matrices.

**Table 2:** Sampled numbers, released numbers, average trigger time and data numbers for three cases.

Different Methods	OB-ETPC${}_{1s}$	OB-ETPC${}_{1.5s}$	TTPC${}_{1s}$	TTPC${}_{1.5s}$	ETC${}_{1s}$	ETC${}_{1.5s}$
Cases	Case 1 and Case 2	Case 1 and Case 3	Case 1	Case 1	Case2	Case 3
Methods	This paper	This paper	[52,53,54]	[52,53,54]	[27,48,49]	[27,48,49]
Sampled numbers	500	500	500	500	500	500
Released numbers	154	154	500	500	143	117
Average trigger time	0.0649 s	0.0649 s	0.02 s	0.02 s	0.0699 s	0.0855 s
Data numbers	114	54	380	130	100	40
